# Early prediction of pathological complete response in rectal cancer: a dynamic immune remodeling hypothesis during neoadjuvant treatment

**DOI:** 10.3389/fonc.2026.1910961

**Published:** 2026-07-31

**Authors:** Serdar Gumus, Cem Kaan Parsak

**Affiliations:** Department of Surgical Oncology, Faculty of Medicine, Çukurova University, Adana, Türkiye

**Keywords:** immune monitoring, neoadjuvant therapy, pathological complete response, rectal cancer, tumor immune microenvironment, tumor regression, watch-and-wait

## Abstract

Achieving pathological complete response (pCR) after neoadjuvant treatment has become one of the most influential outcomes shaping organ preservation strategies, treatment intensification protocols, and long-term oncological decision-making in locally advanced rectal cancer (LARC). Current response assessment, however, remains dependent on delayed structural evaluations — magnetic resonance imaging (MRI), endoscopy, and postoperative pathology — that measure the consequences of tumor regression rather than the biological process generating it. We hypothesize that response to neoadjuvant treatment reflects the temporal dynamics of tumor immune microenvironment (TIME) remodeling: treatment-induced immune activation may precede measurable morphological change, generating immune trajectories that diverge toward complete elimination or toward resistance. We propose that serial, biology-anchored tumor sampling — rather than a single baseline biopsy — can capture these trajectories and support earlier, biologically informed prediction of pCR and sustained clinical complete response (cCR). This article presents the biological rationale for the hypothesis, defines a four-phase mechanistic model of immune remodeling, proposes a measurable and feasibility-conscious biomarker panel with a data-driven scoring approach, states five falsifiable predictions, and outlines a prospective validation framework — including its ethical prerequisites, procedural safety considerations, and analytic handling of treatment-regimen heterogeneity and tumor spatial heterogeneity.

## Introduction

The management of locally advanced rectal cancer (LARC) has undergone substantial evolution over the past two decades. The widespread adoption of neoadjuvant chemoradiotherapy and, more recently, total neoadjuvant therapy (TNT), has significantly improved tumor downstaging and increased rates of pathological complete response (pCR). As organ preservation strategies continue to expand worldwide, the accurate and early identification of patients achieving complete response has become increasingly critical for optimal patient selection (1–5).

Despite these therapeutic advances, current response assessment remains predominantly dependent on structural and anatomical evaluations. Specifically, magnetic resonance imaging (MRI) measures macroscopic changes, endoscopy assesses superficial morphology, and pathological examination confirms tissue response only after definitive surgical intervention. Consequently, these conventional approaches represent a largely retrospective paradigm, measuring what has already occurred rather than identifying biological processes actively unfolding during treatment. This temporal limitation may be particularly relevant in rectal cancer, where treatment-associated biological responses appear to evolve progressively over several weeks (6–9).

Therefore, we propose that dynamic immune remodeling may serve as an earlier and biologically more informative indicator of treatment response than currently available structural assessment approaches (9–11).

## Limitations of the current response paradigm

Contemporary clinical decision-making in rectal cancer remains largely dependent on delayed anatomical endpoints; however, several clinical observations challenge the adequacy of this structural response model. For example, patients demonstrating apparently similar degrees of tumor regression on magnetic resonance imaging (MRI) may ultimately exhibit substantially different pathological outcomes following surgical resection. Likewise, clinical complete response (cCR) does not consistently correspond to pathological complete response (pCR) (8–10).

This diagnostic discordance may arise from tissue fibrosis, treatment-induced inflammation, localized edema, and necrotic remnants, which substantially complicate the interpretation of conventional imaging modalities. Meanwhile, critical biological events continue to evolve throughout the treatment course. The current clinical paradigm largely assumes that reduction in tumor size is synonymous with therapeutic efficacy. However, the biological elimination of viable tumor cells does not necessarily translate into immediate or proportional morphological regression.

This discrepancy may suggest ongoing microscopic biological processes that remain undetected by conventional radiological assessment or surface endoscopic evaluation. These observations suggest that treatment response in rectal cancer may extend beyond structural regression alone and involve dynamic biological processes that evolve over time (9).

## Immunogenic cell death as the initiating event

Radiotherapy and cytotoxic chemotherapy are increasingly recognized not merely as modalities that reduce tumor burden, but also as immunologically active interventions. When administered as neoadjuvant treatment, these modalities may trigger immunogenic cell death (ICD), a process characterized by cell surface calreticulin exposure, extracellular ATP release, and high mobility group box 1 (HMGB1) signaling. Collectively, these danger-associated signals promote dendritic cell activation and enhance tumor antigen presentation, thereby transforming dying tumor cells into potent immune stimulators (11–17).

We hypothesize that treatment-induced tumor destruction initiates a sequential biological cascade beginning with neoantigen exposure and subsequently promoting T-cell priming, localized immune cell recruitment, and progressive immune amplification. This evolving immune cycle may ultimately influence treatment outcomes through sustained antitumor activity and selective elimination of treatment-sensitive tumor clones.

Within this conceptual framework, persistent activation of the immune system may represent one of the major biological drivers underlying complete tumor eradication rather than a passive consequence of tumor regression alone (11, 17). This is further supported by recent single-cell and spatial transcriptomic profiling of paired pre- and post-treatment rectal tumor samples, which has directly visualized treatment-induced remodeling of the tumor microenvironment and its association with therapeutic response (18).

## Dynamic immune remodeling model

We propose that the treatment-associated immune response evolves through four sequential biological phases, each defined by cellular and molecular change within the tumor microenvironment. These phases are conceptual and biologically, not calendrically, defined — their expected correspondence to sampling time points is given in the next section.

### Phase I – baseline immune state

Diagnostic biopsy provides a snapshot of the untreated tumor immune architecture. At this stage, the density and distribution of CD8^+^ cytotoxic T cells, FOXP3^+^ regulatory T cells, CD163^+^ tumor-associated macrophages, and baseline PD-L1 expression are evaluated to characterize the pre-treatment immune landscape (10, 11).

### Phase II – early immune activation

After initiation of neoadjuvant treatment, increased immune cell infiltration may emerge within the tumor microenvironment. In responding tumors, early expansion of CD8^+^ T-cell infiltration, increased granzyme B expression, enhanced antigen presentation, and intensified interferon-mediated immune activation may be observed (13, 16).

### Phase III – immune remodeling and selection

In treatment-sensitive tumors, cytotoxic activity may progressively dominate while resistant clones are selectively eliminated, reflected in a shift toward increased CD8/FOXP3 balance, reduced CD163-associated immunosuppressive dominance, and attenuated regulatory pressure (19–21).

### Phase IV – elimination or resistance

At this stage, patients may diverge into two distinct biological trajectories.

**Route A** – immune elimination: This route is characterized by sustained cytotoxic immune dominance, elevated CD8^+^/FOXP3^+^ balance, increased granzyme B activity, and persistent interferon-associated signaling. Such an immune state may ultimately be associated with pCR or sustained cCR (10, 20, 22).

**Figure 1:**
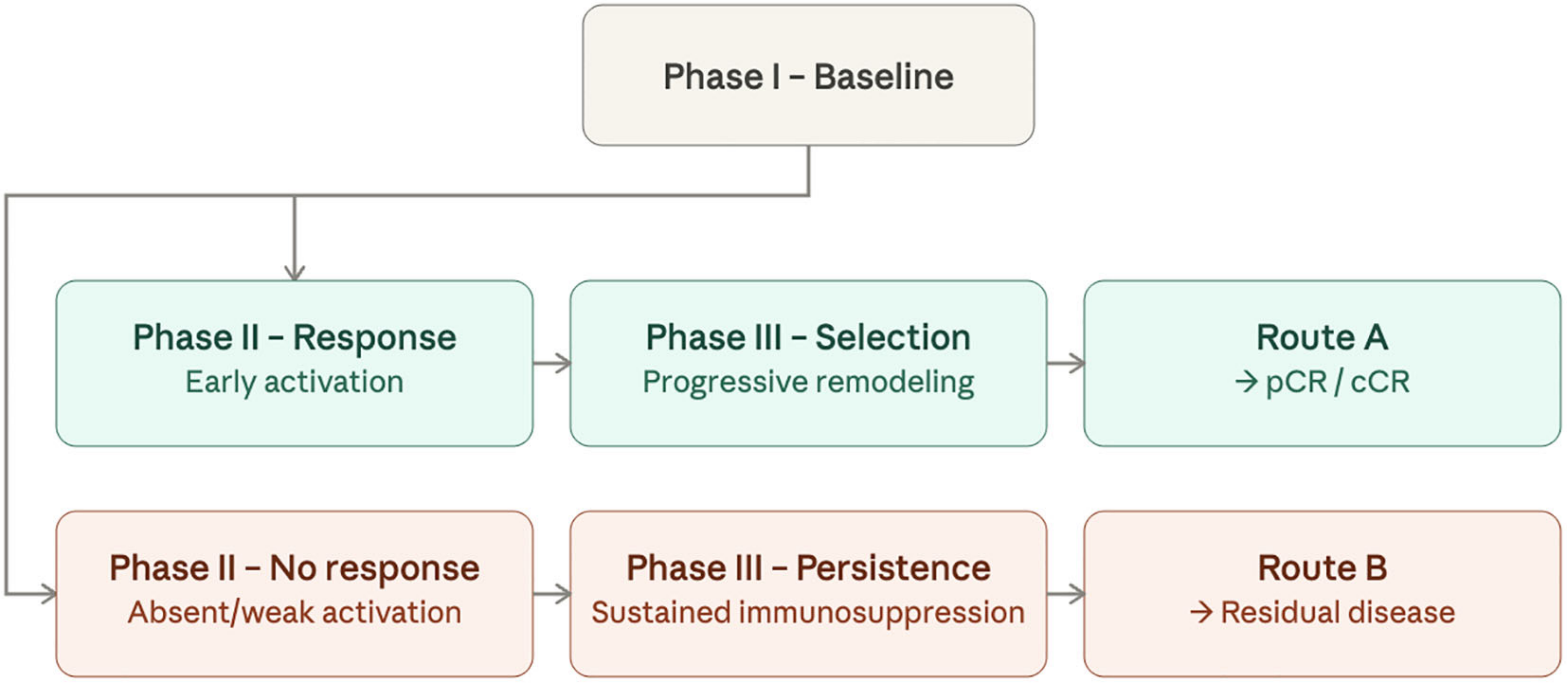
Divergent immune trajectories following neoadjuvant treatment initiation. After baseline assessment (Phase I), tumors follow one of two trajectories. In the responding trajectory (teal), early immune activation (Phase II) progresses to selective remodeling (Phase III), culminating in Route A — sustained cytotoxic dominance associated with pCR/cCR. In the non-responding trajectory (coral), weak early activation progresses to persistent immunosuppression, culminating in Route B — an exhausted, tumor-permissive state associated with residual disease.

**Route B** – immune exhaustion and resistance: This route may be characterized by persistent immunosuppression, increased immune checkpoint activity (particularly the PD-1/PD-L1 axis), the continued presence of regulatory T cells and immunosuppressive macrophages, and the maintenance of a tumor-permissive microenvironment. In selected cases, additional regulatory pathways involving LAG3, TIM3, TIGIT, and TGF-β may further contribute to immune resistance and the persistence of residual disease (10, 11) (**Figure 1**).

## Clinical rationale for serial tumor sampling

A major limitation of current tissue-based biomarkers is their static nature, as a single baseline biopsy cannot capture the dynamic biological transitions that occur throughout treatment regimens spanning several weeks. In contrast, serial tumor sampling may enable clinicians to detect real-time biological changes, including immune activation, early immune suppression, or emerging immune escape mechanisms (10).

Because total neoadjuvant therapy (TNT) is delivered with substantially different sequencing strategies — induction chemotherapy followed by chemoradiotherapy, versus chemoradiotherapy followed by consolidation chemotherapy — a single fixed calendar schedule cannot be applied uniformly across patients without conflating regimen-related timing differences with genuine biological differences in response. We therefore anchor each time point to a regimen-relative biological milestone rather than a universal calendar date, and propose that all analyses be stratified by TNT sequencing strategy (induction- vs. consolidation-based) as a prespecified covariate rather than pooling patients across regimens.

**Figure 2:**
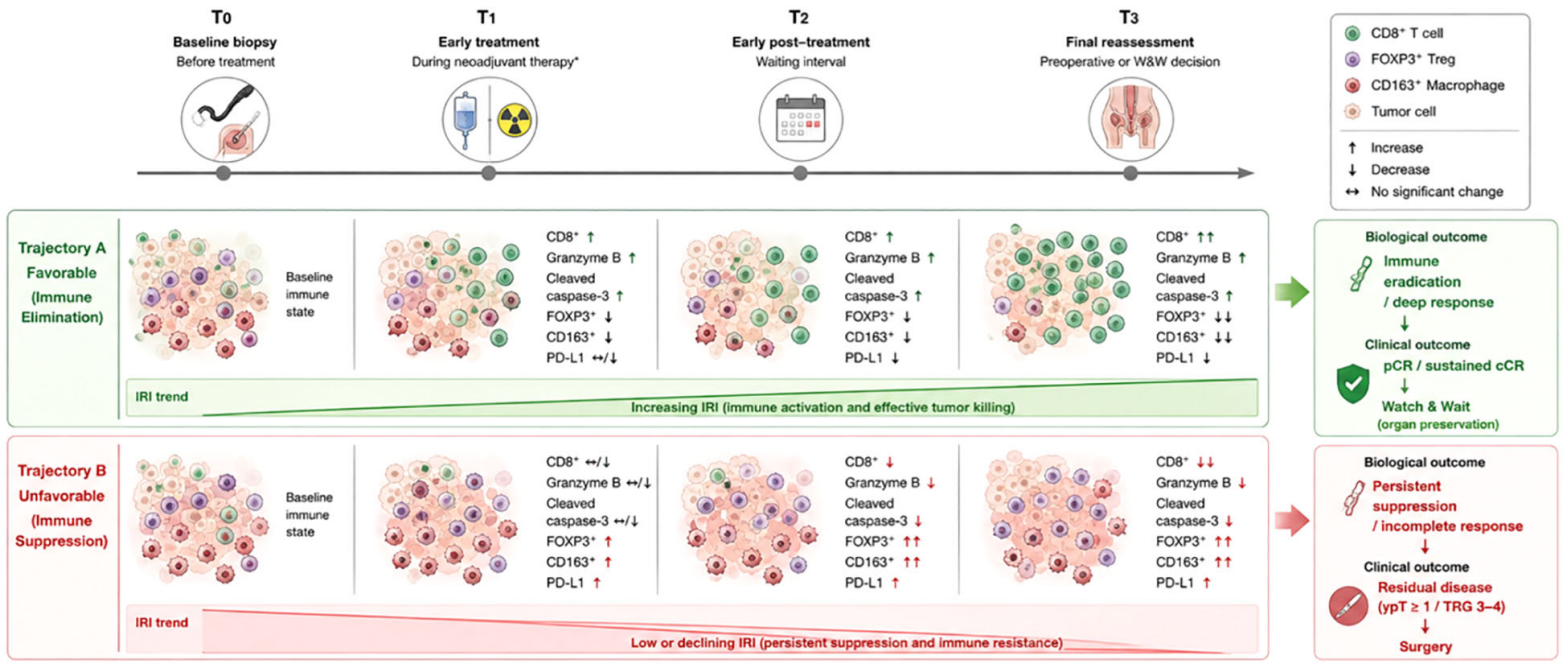
Proposed longitudinal immune remodeling trajectories during total neoadjuvant therapy (TNT): Two hypothetical immune trajectories are illustrated across four biological sampling time points (T0–T3). Trajectory A (immune elimination) is characterized by increasing CD8^+^ T-cell infiltration, granzyme B, cleaved caspase-3, and a rising exploratory Immune Remodeling Index (IRI), accompanied by reduced FOXP3^+^ cells, CD163^+^ macrophages, and PD-L1 expression, ultimately leading to pathological complete response (pCR) or sustained clinical complete response (cCR) and potential eligibility for a watch-and-wait strategy. Trajectory B (persistent immune suppression) demonstrates the opposite pattern and is hypothesized to result in residual viable tumor (e.g., ypT ≥1 or higher Tumor Regression Grade [TRG]) requiring surgical resection. Arrows indicate the expected direction of longitudinal biomarker changes (↑ increase, ↓ decrease, ↔ no significant change). T1 timing is regimen-dependent and corresponds to the early phase of the first-administered treatment modality. The figure is schematic and intended for hypothesis generation rather than representing patient-derived data.

To capture these critical transitions, we propose a four-point temporal framework for evaluation (**Figure 2**; **Table 1**).

**Table 1:** Proposed biology-anchored sampling framework for longitudinal immune monitoring during total neoadjuvant therapy (TNT).

Time point	Definition	Approximate timing	Biological phase captured
T0–Baseline	Diagnostic endoscopic biopsy prior to any therapeutic intervention	Before treatment initiation	Phase I – Baseline immune state
T1–Early treatment*	Biopsy during induction chemotherapy	2–3 weeks after initiation of induction chemotherapy	Phase II — early immune response to chemotherapy alone
	Biopsy during concurrent chemoradiotherapy	2–3 weeks after initiation of chemoradiotherapy	Phase II — early immune response to concurrent chemoradiotherapy
T2–Early post-treatment	Biopsy during the early post-treatment waiting interval	2–3 weeks after completion of all neoadjuvant treatment (i.e., after both chemotherapy and chemoradiotherapy components, regardless of sequencing order)	Phase III – Immune remodeling and selection
T3–Final reassessment	Preoperative reassessment or final evaluation before definitive inclusion in a watch-and-wait strategy	Immediately before surgery, or at the point of definitive non-operative management decision (typically 8–12 weeks after completion of neoadjuvant treatment, per institutional restaging protocol)	Phase IV – Elimination or resistance (outcome ascertainment)

* Because induction- and consolidation-based TNT expose the tumor to mechanistically distinct early treatment components (chemotherapy alone vs. concurrent chemoradiotherapy), T1 is defined separately for each sequencing strategy rather than as a single universal time point. Both variants are intended to capture the same biological phase (Phase II — early immune activation), but the specific treatment exposure driving that activation differs by regimen. This distinction is treated as a prespecified stratification variable in the analysis (see Proposed Prospective Validation) rather than assumed away.

## Four-point temporal framework (T0–T3)

## Biopsy safety and feasibility

Repeated endoscopic tumor sampling, particularly at and after T1–T2 following pelvic radiotherapy, carries recognized procedural risks, including delayed perforation, bleeding, and pelvic sepsis in an irradiated, inflamed rectal wall. These risks must be explicit in any protocol translating this model into practice.

- Proposed exclusion criteria: active radiation proctitis with deep ulceration on prior endoscopy, uncorrected coagulopathy or therapeutic anticoagulation that cannot be safely interrupted, hemodynamic instability, and any endoscopic finding suggestive of impending perforation.
- Proposed safety measures: biopsies restricted to superficial forceps sampling (avoiding deep or large-volume resections), a minimum interval between biopsies, real-time endoscopic risk assessment by an experienced operator, and a prespecified stopping rule if a procedure-related adverse event occurs in the cohort.
- Because T1 is required to test the central hypothesis of early immune activation (Prediction 1), it is not proposed as an optional or omissible time point. Eligibility for the proposed prospective validation study should therefore require that patients be clinically suitable for all four biopsy time points (T0–T3); patients for whom T1 biopsy is contraindicated (see exclusion criteria above) should not be enrolled. Rather than omitting T1 for at-risk patients, risk is managed through the exclusion criteria and safety measures above. This restriction narrows the eligible population and should be explicitly reported as a limitation on generalizability (see Limitations).

## Candidate immune variables and proposed interpretation framework

To enable clinically feasible monitoring of these biological trajectories, we propose a focused and interpretable biomarker panel that can be readily incorporated into routine pathology workflows. Rather than relying on isolated biomarker measurements, the proposed framework emphasizes longitudinal immune remodeling, whereby temporal changes between sequential biopsies are considered more biologically informative than single baseline assessments.

At the cellular level, the density and temporal evolution of CD8^+^ cytotoxic T cells, FOXP3^+^ regulatory T cells, and CD163^+^ tumor-associated macrophages will be evaluated. An increase in CD8^+^ T-cell density over time is hypothesized to reflect enhanced antitumor cytotoxic immunity, whereas persistent or increasing FOXP3^+^ and CD163^+^ cell populations may indicate sustained immunosuppressive activity and macrophage-mediated immune resistance within the tumor microenvironment (10–17).

At the functional level, granzyme B and PD-L1 expression will be assessed. Dynamic increases in granzyme B expression are expected to reflect enhanced cytotoxic activity mediated by activated T cells and natural killer (NK) cells, whereas persistent PD-L1 expression may represent adaptive immune resistance and continued immune checkpoint activation (23).

At the level of tumor cell death, cleaved caspase-3 may serve as a complementary indicator of active apoptosis, providing biological evidence that immune activation is accompanied by effective tumor-cell elimination rather than immune-cell infiltration alone (24).

Importantly, the proposed biomarker panel is intended to be interpreted longitudinally rather than through absolute biomarker values. We hypothesize that treatment response is better characterized by the direction and magnitude of immune remodeling over time than by isolated measurements obtained at a single time point. Accordingly, dynamic variables such as ΔCD8 density, ΔCD8/FOXP3 ratio, ΔCD8/CD163 ratio, Δgranzyme B expression, ΔPD-L1 expression, and Δcleaved caspase-3 are proposed as candidate indicators of treatment-induced immune remodeling.

As a provisional, hypothesis-generating starting point, the IRI may be calculated as an equal-weighted composite of standardized (z-score) longitudinal changes in markers of cytotoxic-to-suppressive balance and tumor-cell death: IRI = z(ΔCD8/FOXP3) + z(ΔCD8/CD163) + z(Δgranzyme B) − z(ΔPD-L1) + z(Δcleaved caspase-3).

Raw CD8^+^ density is not entered separately, since it already contributes to both ratio terms; including it a third time would overweight a single biological signal. Equal weighting is used only as a default starting point — the relative contribution of each term, and whether any term should be dropped or regularized (e.g., via LASSO), is expected to be re-estimated empirically against pCR/sustained cCR during prospective validation. This formula should not be treated as fixed or validated.

As noted above, the IRI is designed to summarize the overall balance between cytotoxic immune activation, immune suppression, macrophage polarization, immune checkpoint signaling, and tumor-cell apoptosis. Because no validated quantitative thresholds currently exist for dynamic immune remodeling in rectal cancer, predefined universal cutoff values are intentionally not proposed. Instead, optimal weighting of individual biomarkers and clinically relevant cutoff values should be determined empirically during prospective validation using receiver operating characteristic (ROC) analysis against pathological complete response (pCR) and sustained clinical complete response (cCR), followed by independent external validation before clinical implementation. (**Figure 2**; **Table 2**).

**Table 2:** Candidate biomarker panel and hypothesized direction of favorable longitudinal change.

Biomarker	Expected favorable change	Biological interpretation
CD8^+^ T cells	↑	Enhanced cytotoxic antitumor immune activity
CD8/FOXP3 ratio	↑	Reduced regulatory immune suppression
CD8/CD163 ratio	↑	Reduced macrophage-mediated immunosuppression
Granzyme B	↑	Increased cytotoxic tumor-cell killing
PD-L1	↓ or normalization	Reduced adaptive immune resistance
Cleaved caspase-3	↑	Increased tumor-cell apoptosis

Changes refer to the direction of longitudinal (Δ) variation between sequential biopsies (T0–T3), not absolute values at a single time point. No validated quantitative thresholds currently exist; optimal cutoffs are to be determined empirically during prospective validation (see Candidate Immune Variables and Proposed Interpretation Framework). CD8^+^ T-cell density is monitored independently and also contributes to the CD8/FOXP3 and CD8/CD163 ratio terms above; it is not entered a third time into the IRI formula to avoid overweighting a single biological signal (see Candidate Immune Variables and Proposed Interpretation Framework). CD8, cluster of differentiation 8; FOXP3, forkhead box P3; CD163, cluster of differentiation 163; PD-L1, programmed death-ligand 1.

As a provisional approach informed by percentile-based stratification used in related single-timepoint immune biomarkers in rectal cancer (10), Δ variables may initially be stratified into tertiles (lowest, middle, highest third of the cohort distribution) rather than fixed percentage cutoffs, with the highest tertile of ΔCD8-related measures and the lowest tertile of ΔPD-L1 provisionally defining ‘favorable’ longitudinal change. Fixed numerical thresholds (e.g., percentage-based cutoffs) are deliberately not proposed at this stage, given the absence of validated longitudinal cutoffs in the literature; tertile-based stratification is intended only as a placeholder for ROC-based optimization during prospective validation.

Overall, this framework aims to provide a biologically integrated interpretation of treatment response by capturing the dynamic interaction between immune activation, immune suppression, and tumor-cell death, thereby extending response assessment beyond conventional structural and anatomical evaluation.

## Proposed methodological specifications

Biopsy specimens will be obtained using standard endoscopic biopsy forceps, with 2–3 passes per anatomical region (tumor center and invasive margin; see Limitations) at each time point, fixed in formalin and paraffin-embedded per standard pathology protocols (10, 25). Immunohistochemical staining will use commercially validated antibody clones for CD8, FOXP3, CD163, granzyme B, PD-L1, and cleaved caspase-3, with antigen retrieval, dilution, and detection protocols standardized across all time points and validated on positive control tissue prior to cohort-wide application (21, 23); specific clones and dilutions will be finalized and reported in the validation study protocol. Quantification will be performed using digital image analysis (cells/mm² for cellular markers; H-score or percentage positivity for PD-L1) to minimize inter-observer variability (10), with a subset independently scored by two pathologists to establish reproducibility (e.g., intraclass correlation coefficient).

## Testable predictions

To facilitate prospective evaluation of the proposed biological framework, we formulate five specific and falsifiable hypotheses describing the expected relationships between longitudinal immune remodeling and clinically relevant treatment outcomes (**Figure 2**). These predictions are intended to guide future validation studies rather than prescribe a definitive analytical protocol.

### Prediction 1 – early cytotoxic activation predicts treatment response

Immune activation emerging during the early phase of neoadjuvant treatment may represent one of the earliest biological indicators of treatment success. Specifically, patients demonstrating an early increase in intratumoral CD8^+^ cytotoxic T-cell infiltration, cytotoxic activity, or an increase in the Immune Remodeling Index (IRI) between T0 and T1 are hypothesized to have a higher probability of achieving pathological complete response (pCR) or sustained clinical complete response (cCR) than patients without such immune activation (10, 25).

This concept emphasizes not the baseline immune status of the tumor, but rather its capacity to undergo immune transformation under therapeutic pressure. The hypothesis would be considered unsupported if prospective studies demonstrate no significant association between early immune activation and treatment response after adjustment for relevant clinical confounders.

### Prediction 2 – persistent immune suppression may underlie residual disease

Persistent immunosuppressive remodeling during the waiting interval may represent one of the principal biological mechanisms underlying incomplete tumor regression and residual disease (11, 12). Patients demonstrating sustained FOXP3^+^ regulatory T-cell activity, persistent CD163^+^ macrophage predominance, continued PD-L1 expression, or persistently low IRI values between T1 and T2 are hypothesized to have a higher likelihood of residual viable tumor than patients exhibiting progressive immune activation.

This hypothesis would be considered unsupported if longitudinal immunosuppressive trajectories show no significant association with pathological residual disease.

### Prediction 3 – temporal immune remodeling may complement current restaging approaches

Longitudinal assessment of immune remodeling may provide biological information beyond conventional imaging-based response assessment (10). We hypothesize that integrating serial immune remodeling variables, including the IRI, with standard MRI-based restaging will improve prediction of pathological complete response or sustained clinical complete response compared with imaging assessment alone.

This hypothesis would be considered unsupported if the addition of longitudinal immune variables fails to improve predictive performance in prospective validation studies.

### Prediction 4 – immune signatures may guide organ preservation strategies

Distinct immune trajectories emerging during the post-treatment waiting interval may help identify patients most likely to achieve durable clinical response and therefore represent appropriate candidates for organ preservation (10, 11, 26). Among patients managed with a watch-and-wait strategy, those demonstrating a sustained immune elimination signature between T2 and T3 are hypothesized to have lower rates of local regrowth than patients exhibiting persistent immunosuppressive trajectories.

This hypothesis would be considered unsupported if long-term oncological outcomes do not differ according to longitudinal immune trajectory.

### Prediction 5 – immune trajectories may enable biology-guided adaptive treatment strategies

The dynamic evolution of the tumor immune microenvironment may provide a biological foundation for future adaptive treatment strategies. We hypothesize that longitudinal immune trajectories could ultimately inform individualized treatment intensity, optimal waiting duration, or the selection of additional biological interventions, replacing fixed chronological algorithms with biology-guided decision-making.

Future prospective studies may therefore evaluate whether treatment strategies adapted according to immune remodeling perform better than conventional fixed treatment schedules. The purpose of this prediction is not to establish an immediate clinical recommendation but to generate a testable hypothesis for future interventional trials.

### Overall concept

Within this framework, tumor response is conceptualized not as a static endpoint but as a dynamic, time-dependent, and biologically measurable process. Consequently, serial immune remodeling may provide clinically actionable information that complements anatomical assessment and supports more individualized treatment strategies in locally advanced rectal cancer.

## Proposed prospective validation

A prospective validation framework is envisioned in patients with LARC undergoing TNT, using serial endoscopic sampling at T0–T3 as defined above, with regimen-stratified analysis (induction- vs. consolidation-based TNT) and adjustment for MSI/dMMR status as a prespecified covariate rather than an afterthought.

Given that T1 reflects different treatment exposures across TNT sequencing strategies, all comparisons involving T1 (including Prediction 1) will be performed within each regimen stratum separately, with pooled analysis reported only as a secondary, exploratory step using regimen as a covariate.

This study, as proposed, has not been conducted, and no human data or biological samples have been collected in the preparation of this hypothesis article. Any prospective study implementing this framework will require institutional review board (or equivalent ethics committee) approval, written informed consent from all participants, prospective trial registration, and a formal safety-monitoring plan (see Biopsy Safety and Feasibility) prior to enrollment of any patient or collection of any sample.

Baseline biopsy (T0) represents pretreatment immune architecture; T1 captures emerging early activation; T2 (waiting interval) captures signatures associated with elimination or resistance; T3 provides the final biological state before the definitive clinical decision. In patients undergoing surgery, biological trajectories will be compared against pCR (ypT0N0) as the primary reference standard. In patients entering watch-and-wait, trajectories will instead be compared against sustained clinical complete response at a prespecified follow-up interval (e.g., 2 years without local regrowth) as a distinct primary endpoint for that cohort — these two endpoints will be analyzed separately and not pooled, since they reflect different biological confirmations of response (pathological absence of tumor vs. sustained absence of clinical regrowth).

Sample size will be calculated based on the expected effect size for Prediction 1 (e.g., an odds ratio derived from comparable Immunoscore-based studies (10)), targeting 80% power at a two-sided alpha of 0.05. Longitudinal biomarker trajectories will be analyzed using mixed-effects models to account for repeated measures within patients, and multiple comparisons across the five predictions will be addressed using a prespecified false discovery rate control (e.g., the Benjamini-Hochberg procedure) (27).

In the longer term, such longitudinal assessment may contribute to biologically informed decision-support tools that complement current restaging approaches, contingent on successful prospective validation of the predictions above.

## Potential clinical implications

Evaluating tumor response not solely by structural regression but by immune remodeling may open the way to a more dynamic and biologically informed decision-making model in rectal cancer management.

A real-time immune monitoring approach may ultimately enable the incorporation of treatment-associated biological changes into clinical decision-making. Patients demonstrating pronounced early immune activation may have a higher probability of achieving complete response, whereas persistent immune suppression may identify individuals who could benefit from alternative therapeutic strategies or closer reassessment.

Similarly, evaluation of immune profiles emerging during the post-treatment waiting interval may provide additional biological information regarding which patients are more suitable for organ preservation strategies and which may benefit from prioritization of surgical intervention.

In current clinical practice, surgical timing following neoadjuvant treatment is largely determined according to predefined chronological intervals. However, observations suggesting increasing complete response rates with prolonged waiting periods imply that treatment effects may not represent a static event completed at a single time point, but rather the consequence of dynamic biological processes evolving over several weeks (2, 5).

Continued immunogenic cell death, sustained antigen presentation, cytotoxic T-cell activity, and ongoing remodeling of the tumor microenvironment after treatment completion may support the possibility that tumor elimination continues beyond the end of active therapy.

Accordingly, the optimal waiting interval in the future may be considered not as a universal chronological window applicable to all patients, but as an adaptive process guided by individual tumor biology. Patients demonstrating early immune elimination signatures may benefit from prolonged observation with a potentially greater probability of complete response, whereas delaying surgery in tumors with persistent immunosuppressive features may offer limited additional benefit.

In other words, the waiting interval may ultimately be determined immunologically rather than chronologically.

In the long term, this approach may contribute to the development of biologically informed decision-support tools that complement current restaging methods, reduce unnecessary treatment burden, enable more precise implementation of organ preservation strategies, and support the transition toward increasingly personalized models of rectal cancer management.

## Limitations

Although this model provides a conceptual framework for the biological dynamics of treatment response, several important limitations remain.

- Regimen heterogeneity: despite biology-anchored time points, induction- and consolidation-based TNT may still generate qualitatively distinct immune kinetics that are not fully captured by the proposed anchoring scheme; regimen-stratified analysis mitigates but does not eliminate this issue.
- Generalizability constraint from mandatory T1 sampling: Distinct from the procedural feasibility concerns below, mandatory T1 eligibility also raises a selection-related limitation: because eligibility requires clinical suitability for all four biopsy time points (T0–T3), the study population may systematically exclude patients at higher bleeding, perforation, or comorbidity risk (see Biopsy Safety and Feasibility). This may limit the generalizability of study findings — including Prediction 1 — to the broader, more heterogeneous LARC population encountered in routine clinical practice.
- Procedural feasibility and safety: serial endoscopic sampling carries bleeding, perforation, and sepsis risk, particularly after radiotherapy (see Biopsy Safety and Feasibility). Patient compliance with up to four biopsy time points may be limited in practice, and studies should report attrition and procedure-related adverse events transparently.
- Spatial heterogeneity: rectal tumors show substantial immune variation between center, invasive margin, and stromal compartments. A single-region biopsy at each time point risks attributing sampling variation to biological change. We propose a minimum of two to three biopsies per time point (tumor center and invasive margin) and recommend integrating digital pathology and spatial analysis tools, informed by recent rectal- and colorectal-cancer-specific spatial transcriptomic work describing distinct TME landscapes predictive of immunotherapy response (28, 29), to formally quantify and adjust for this variability rather than treating it solely as a caveat.
- Molecular confounders: MSI/dMMR status and treatment-induced systemic lymphopenia can independently influence both baseline and treatment-associated TIME features; these must be measured and adjusted for, not assumed away.
- Interpretation of treatment-related tissue change: post-treatment inflammation, fibrosis, necrosis, and remodeling may complicate interpretation of immune cell densities; temporal change should be interpreted in biological context rather than through absolute counts alone.
- Analytic infrastructure: integrated interpretation of cellular, functional, spatial, and molecular data will likely require digital imaging platforms and high-dimensional bioinformatic methods beyond routine pathology capacity, which should be budgeted for in any validation study.
- Complementary less-invasive biomarkers: circulating tumor DNA (ctDNA), circulating immune profiling, exosomal analysis, and imaging-derived radiomic-immunologic signatures (26) may serve as complementary or partial alternatives to repeated tissue sampling and should be evaluated in parallel, both for scientific triangulation and as a risk-mitigation strategy for patients unable to safely undergo repeated biopsy.

Overall, the immune remodeling model remains a biological framework awaiting prospective validation and will likely require enrichment through multimodal, regimen-stratified, and less invasive approaches before clinical applicability can be established.

## Conclusion

Response to neoadjuvant treatment in rectal cancer may represent not merely a consequence of delayed structural regression, but also the clinical manifestation of a dynamic process of immune remodeling and tumor elimination evolving over time.

The proposed serial immune monitoring framework may offer a complementary approach for evaluating the biological behavior of tumors throughout treatment. Integrating immune activation, immune suppression, and real-time tumor cell death assessment may provide additional biological insight into the probability of complete response, the risk of residual disease, and the potential for organ preservation beyond conventional restaging approaches.

The clinical value of the proposed model has not yet been established and requires prospective validation. In particular, studies investigating the relationship between real-time immune remodeling patterns and pathological complete response, sustained clinical complete response, and long-term oncological outcomes will be essential to determine the clinical applicability of this conceptual framework.

In the longer term, systematic monitoring of immune remodeling may provide a theoretical foundation for moving from imaging-driven static decision models toward more adaptive and biologically informed treatment strategies in rectal cancer management.

## Data Availability

The original contributions presented in the study are included in the article/supplementary material. Further inquiries can be directed to the corresponding author.
